# PPARs and Microbiota in Skeletal Muscle Health and Wasting

**DOI:** 10.3390/ijms21218056

**Published:** 2020-10-29

**Authors:** Ravikumar Manickam, Kalina Duszka, Walter Wahli

**Affiliations:** 1 Department of Pharmaceutical Sciences, University of South Florida, 12901 Bruce B. Downs Blvd., Tampa, FL 33612, USA; ravikumarm@usf.edu; 2 Department of Nutritional Sciences, University of Vienna, Althanstrasse 14, 1090 Vienna, Austria; kalina.duszka@univie.ac.at; 3 Center for Integrative Genomics, University of Lausanne, Le Génopode, CH-1015 Lausanne, Switzerland; 4 Toxalim, INRAE, Chemin de Tournefeuille 180, F-31027 Toulouse, France; 5 Lee Kong Chian School of Medicine, Nanyang Technological University Singapore, Clinical Sciences Building, 11 Mandalay Road, Singapore 308232, Singapore

**Keywords:** PPAR, muscle, microbiota, gut, metabolism

## Abstract

Skeletal muscle is a major metabolic organ that uses mostly glucose and lipids for energy production and has the capacity to remodel itself in response to exercise and fasting. Skeletal muscle wasting occurs in many diseases and during aging. Muscle wasting is often accompanied by chronic low-grade inflammation associated to inter- and intra-muscular fat deposition. During aging, muscle wasting is advanced due to increased movement disorders, as a result of restricted physical exercise, frailty, and the pain associated with arthritis. Muscle atrophy is characterized by increased protein degradation, where the ubiquitin-proteasomal and autophagy-lysosomal pathways, atrogenes, and growth factor signaling all play an important role. Peroxisome proliferator-activated receptors (PPARs) are members of the nuclear receptor family of transcription factors, which are activated by fatty acids and their derivatives. PPARs regulate genes that are involved in development, metabolism, inflammation, and many cellular processes in different organs. PPARs are also expressed in muscle and exert pleiotropic specialized responses upon activation by their ligands. There are three PPAR isotypes, viz., PPARα, -β/δ, and -γ. The expression of PPARα is high in tissues with effective fatty acid catabolism, including skeletal muscle. PPARβ/δ is expressed more ubiquitously and is the predominant isotype in skeletal muscle. It is involved in energy metabolism, mitochondrial biogenesis, and fiber-type switching. The expression of PPARγ is high in adipocytes, but it is also implicated in lipid deposition in muscle and other organs. Collectively, all three PPAR isotypes have a major impact on muscle homeostasis either directly or indirectly. Furthermore, reciprocal interactions have been found between PPARs and the gut microbiota along the gut–muscle axis in both health and disease. Herein, we review functions of PPARs in skeletal muscle and their interaction with the gut microbiota in the context of muscle wasting.

## 1. Introduction

Skeletal muscle makes up to 40 percent of the total body weight in healthy humans [1]. It is a striated muscle, which is under the voluntary control of the somatic nervous system. The skeletal muscles are attached to bones via strong connective tissues called tendons or to other muscles or tissues, andfacilitates support and movement. Skeletal muscle is also a massive metabolic organ that utilizes the majority of the available glucose for adenosine triphosphate (ATP) production through insulin-mediated glucose uptake, and stores the excess glucose as glycogen. It is also involved in fatty acid (FA) oxidation for energy production during exercise, fasting, and insulin resistance [2,3,4]. Deregulation of muscle metabolism can result in metabolic syndrome, obesity, and diabetes, which predispose to various other illnesses, including cardiovascular diseases [5,6].

**Figure 1:**
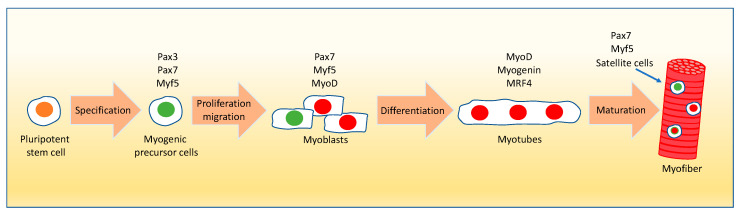
Myogenic lineage in myofiber formation. The myogenic precursor cells (Pax3/Pax7/Myf5 positive) are derived from pluripotent stem cells of the dermomyotome compartment of the somites during embryogenesis and undergo multiple waves of division followed by migration for the formation of myofibers in different parts of the body. The primary myofibers arise mostly from Pax3 positive myogenic progenitor cells, whereas the secondary myofibers are formed from the Pax7 positive myogenic progenitors using the primary myofibers as scaffold. Subsequent to activation and proliferation, the myogenic precursor cells transform into myoblasts expressing Pax7, Myf5, and MyoD. Myf5 acts alongside the expression of paired box genes, which is followed by the expression of MyoD, a downstream effector of myogenesis. During differentiation, the committed myoblasts, also known as myocytes expressing MyoD, exit the cell cycle, and fuse to form the multinucleated myotubes upon the induction of Myogenin and MRF4 genes. During both myoblast proliferation and differentiation some cells maintain high levels of Pax7 expression and self-renew to maintain tissue homeostasis (satellite cells pool). The mature myotubes turn into myofibers upon the expression of various structural proteins such as myosin light and heavy chains and myocyte enhancer factor 2c. Myonuclei—red; stem cell nuclei—green. The blue arrow points to a satellite cell located on the myofiber. Pax3/7: paired box 3/7; Myf5: myogenic factor 5; MyoD: myogenic determination factor D1; MRF4: muscle regulatory factor 4.

The skeletal muscle consists of oxidative and glycolytic fibers that differ in their contractile properties [7,8]. Moreover, it is made by different cell populations, including myocytes, stem cells, and fibroblasts. It also contains nerve endings and blood vessels [8,9]. The process of myogenesis is tightly controlled by a complex series of spatiotemporal signaling cascades. It originates from muscle precursor cells during embryogenesis in the dermomyotome compartment of the somites [10,11,12]. These cells express the paired box proteins Pax3, Pax7, and myogenic factor 5 (Myf5) transcription factors (Figure 1). The muscle precursor cells de-epithelialize by the interaction of the scatter factor/hepatocyte growth factor (SF/HGF) with its receptor c-met. The long-range migration of these cells to form the various muscles in the entire body also requires this receptor–ligand interaction [13,14]. Throughout development, multiple waves of muscle precursor cells, called myoblasts, originate from the skeletal muscle progenitor cells. During both mouse embryogenesis and neonatal life, i.e., the initial 4 weeks after birth, increased proliferation (hyperplasia) of myoblasts takes place [15,16,17,18]. During postnatal muscle growth, the proliferating myoblasts fuse to form the differentiated multinucleated myotubes resulting in increased muscle fiber size (hypertrophy) (Figure 1). Myocytes express Myf5, the myogenic determination factor MyoD, Myogenin, and the myogenic regulatory factor MRF4. Myf5 and MyoD are myogenic determination factors that belong to the helix-loop-helix superfamily of transcription factors, whereas Myogenin and MRF4 are myogenic differentiation factors [13,14,19,20]. These myogenic transcription factors bind to the promoter of their target genes, such as the myocyte enhancer factor *Mef2*, and peroxisome proliferator-activated receptor (PPAR) gamma coactivators *Pgc-1α* and *Pgc-1β* to regulate their expression levels [17]. During myogenesis, a portion of the progenitor cell population does not differentiate, but self-renews to maintain the muscle stem cell pool for tissue homeostasis. This self-renewal process is accompanied by a high level of Pax7 expression in these cells [21]. During postnatal growth, adult muscle stem cells are referred to as satellite cells because of their location underneath the basal lamina of myofibers (Figure 1). The satellite cells are thought to be derived from the progenitor cells originating from the dermomyotome cell population [11,22]. They retain an uncommitted state in contrast to the more committed myogenic progenitors [22,23,24]. Ultimately, these satellite cells are responsible for postnatal muscle growth and maintenance, repair, and regeneration in the aging muscle and after muscle injury [25,26,27,28,29,30,31,32,33]. However, the number of skeletal muscle satellite cells decreases during aging, which causes a loss of muscle mass due to a reduction of regenerative capacity and function [34,35,36,37]. Various secreted signaling molecules from the muscle and its surrounding tissues not only induce myogenesis but also influence cellular processes, such as muscle cell proliferation and differentiation [38]. Thus, the proper coordination of the molecular events starting in stem cells is crucial for muscle development, repair, and regeneration, as well as function in postnatal growth. The mechanisms of satellite cell activation and self-renewal are not yet fully understood. However, various growth factors and signaling pathways have been implicated in these processes during skeletal muscle regeneration [39,40,41,42]. The already-mentioned paired box transcription factors Pax3 and Pax7 play an essential role in the processes of early specification, migration, myogenic differentiation, and skeletal muscle formation [21,43,44]. Pax7 is the most consistently expressed satellite cell marker across species and different muscles. Pax7-null mice display a complete absence of satellite cells, suggesting that Pax7 is a crucial factor for satellite cell biogenesis and survival [45,46]. Overexpression of Pax7 inhibits cell cycle progression and myogenesis by downregulating MyoD. Pax7 also plays a major role in satellite cell self-renewal [21]. During routine maintenance of tissue homeostasis, satellite cells are recruited individually for the localized repair of subtle injuries and self-renew to maintain tissue homeostasis. Upon muscle injury, the quiescent satellite cells expressing high levels of Pax7 and Myf5 get activated and proliferate with stimulation of MyoD expression. The proliferating myoblasts then fuse to differentiate into new multinucleated myotubes expressing Myogenin, which are key players in muscle repair.

Skeletal muscle fiber has high remodeling plasticity on demand, but is also prone to deterioration. Muscle mass is lost in various genetic abnormalities that are commonly observed in several myopathies and muscular dystrophies to varying degrees [47]. Moreover, metabolic syndrome and its manifestations (obesity and diabetes), and other diseases, including cancer cachexia, heart, respiratory and kidney failures, severe burns, and sepsis also trigger muscle mass reduction [48,49]. During aging, the muscle mass decreases gradually beginning at the age of approximately 40 years in humans. This process, referred to as sarcopenia, which also comprises a decrease in muscle stem cell number and regenerative capacity, is often accompanied by osteopenia/osteoporosis [34,35,36,37].

The aim of this review is to summarize the roles PPARs and their crosstalk with microbiota in skeletal muscle health and wasting. Understanding these roles in skeletal muscle pathophysiology and organ–organ crosstalk, including the gut–microbiota interactions would pave the way for future therapeutic and lifestyle interventions in muscle diseases.

## 2. The Peroxisome Proliferator-Activated Receptors (PPARs)

PPARs belong to the nuclear receptor superfamily of transcription factors, which is comprised of 48 and 49 members in humans and mice, respectively [8,50,51,52]. Nuclear receptors are activated by specific natural ligands such as lipids, retinoids, steroids, and thyroid hormones. They are also the targets for synthetic therapeutic compounds. These receptors and their ligands regulate a multitude of diverse functions in all organs during development and the whole lifespan, and in various pathophysiological conditions, such as inflammation and metabolic diseases [8,52,53]. Many nuclear receptors are found to be expressed in skeletal muscle, including PPARs [54]. The PPAR subfamily comprises three related isotypes, PPARα (NR1C-1), PPARβ/δ (NR1C-2), and PPARγ (NR1C-3), produced by three different genes located on different chromosomes in vertebrates [8,54,55,56,57,58,59,60]. The whole subfamily was first identified in *Xenopus laevis*. Based on the sequence similarities with PPARs from mammals, they were named PPARα, PPARβ/δ, and PPARγ [59,60,61,62]. PPARs heterodimerize with retinoid X receptors (RXRs) and upon ligand activation bind to peroxisome proliferator response elements (PPRE) in the regulatory regions of their target genes [8,63]. PPARs either activate or repress gene expression by recruiting co-activators or co-repressors, respectively. The activity of PPARs is also modulated through phosphorylation, SUMOylation, ubiquitination, and interactions with coregulators as mentioned above [8,58,64,65,66,67,68,69].

PPARs are often co-expressed in different tissues, including skeletal muscle at variable levels [8]. PPARα is highly expressed in the liver, heart, brown adipose tissue, and kidney; it is also found in skeletal muscle. Among other roles, it has an important function in fatty acid catabolism [70,71,72,73,74,75]. PPARα regulates the peroxisomal and mitochondrial β-oxidation, and the microsomal ω-oxidation of fatty acids. It also participates in glucose metabolism, and is key in the control of energy expenditure and inflammation [55,73,74]. PPARβ/δ is ubiquitously expressed, but at different levels, in various tissues and has several functions. It is the predominant PPAR isotype in skeletal muscle, and is also expressed in muscle satellite cells [8,67]. It is involved in lipid and glucose metabolism, energy expenditure, inflammation, tissue repair and regeneration, and myofiber type switch associated with physical exercise [68,69,75,76,77,78]. There are two isoforms of PPARγ, viz., PPARγ1 and PPARγ2 [79]. PPARγ1 is highly expressed in adipocytes and is found to be expressed at variable levels in other tissues such as the liver and colon for example. PPARγ2 is predominantly expressed in adipose tissue and plays a major role in adipogenesis and triglyceride storage. One of the main functions of PPARγ is the deposition of fat in several organs, including skeletal muscle [79,80,81].

Over the years, the pharmacological activation of PPARs has received much attention, not least because of their hypolipidemic and anti-diabetic effects. Well-known activators of the fibrate and thiazolidinedione classes of compounds, as well as dual- and pan-agonists, are used in clinics or are in development for the treatment of conditions such as non-alcoholic fatty liver disease (NAFLD). These compounds and their beneficial as well as their potential adverse effects have recently been extensively reviewed [82,83,84,85,86,87] and therefore will not be further discussed here.

## 3. Roles of PPARs in Muscle

**Figure 2:**
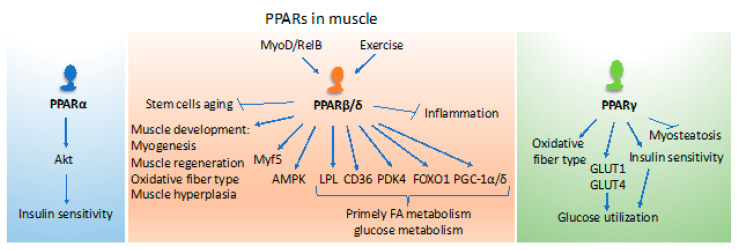
The roles of PPARs in the muscle. PPARα impacts insulin sensitivity in muscles. PPARβ/δ plays multiple roles in muscle development, function, metabolism, and inflammation. The function of PPARγ is mainly connected with metabolism and energy utilization. However, it also contributes to changes in muscle fiber type and inhibits myosteatosis. The list of depicted functions is not exhaustive and relates to the content of the review. PPAR: peroxisome proliferator-activated receptor; Akt: serine/threonine kinase also known as Protein Kinase B (PKB); MyoD: myoblast determination protein 1; Myf5: myogenic factor 5; RelB: a member of the NF-κB family; AMPK: adenosine monophosphate-activated protein; LPL: lipoprotein lipase; CD36: cluster of differentiation 36, also known as fatty acid translocase (FAT); PDK4: pyruvate dehydrogenase kinase 4; FOXO1: forkhead box protein O1; PGC-1: peroxisome proliferator-activated receptor gamma coactivator 1; GLUT1: glucose transporter 1; GLUT4: glucose transporter 4.

PPARs are key players in the control of different aspects of metabolism that are needed for muscle activities and thermoregulation [88,89] (Figure 2). Both PPARα and PPARβ/δ regulate genes coding for proteins participating in fatty acid uptake and mitochondrial β-oxidation, such as the cluster of differentiation 36 (CD36), lipoprotein lipase (LPL), fatty acid-binding protein 3 (FABP3), carnitine palmitoyltransferase 1 (CPT1), and stearoyl-CoenzymeA desaturase (SCD). However, PPARβ/δ has emerged as a master regulator of fatty acid catabolism in muscle and has, therefore, received much attention in muscle biology, as will be discussed below. In the fed state, most of the circulating glucose is utilized by skeletal muscle for energy (ATP) production under the control of insulin signaling. Glucose excess is used for lipid synthesis or stored as glycogen. Although PPARα is involved in de novo lipid synthesis and storage of fatty acids as triglycerides, PPARγ is key for the deposition of fat in muscle tissues. During fasting, both PPARα and PPARβ/δ regulate the cellular uptake and use of free fatty acids as an alternate energy source for ATP production through the mitochondrial β-oxidation pathway [8,54,88,89]. Several observations suggest at least partial interlinking and overlapping roles of the PPAR isotypes in muscle.

Transgenic mice overexpressing PPARα in muscles develop glucose intolerance, and are insulin resistant even though they are protected from diet-induced obesity. This lean phenotype is accompanied by decreased 5′ adenosine monophosphate-activated protein kinase (AMPK) activity as well as reduced glucose transporter GLUT4, MEF2A, and PGC1α expression [90]. In line with these observations, diet-induced insulin resistance is prevented in PPARα knockout (KO) mice despite being obese and with no alterations in the AMPK activity when compared to wild-type mice [90]. PPARα agonists are used to treat patients with metabolic syndrome to improve lipid metabolism and insulin sensitivity [89]. However, the direct effects of these drugs on the skeletal muscle are not known. Recently, it was reported that bezafibrate treatment of streptozotocin-injected diabetic mice that mimic type 1 diabetes improves skeletal muscle insulin sensitivity through the serine/threonine kinase Akt phosphorylation and signaling, which may also be due to the improved whole-body insulin sensitivity [91] (Figure 2). Since bezafibrate is a pan-PPAR agonist, the two other PPAR isotypes may potentially also participate in this effect as the expression level of all three PPARs was found to be decreased with streptozotocin treatment [91]. In a non-small cell lung cancer mouse model associated with muscle wasting that mimic the human cancer cachexia syndrome, administration of fenofibrate restores the loss of muscle mass and body weight [92]. Furthermore, the expression level of PPARα is increased in the skeletal muscle of humans undergoing endurance exercise [93,94].

PPARβ/δ in muscle has been studied in various experimental systems, not least in cell cultures, mainly in vitro models of mouse and rat myoblasts, and human primary myotubes cultures treated with either fatty acids or synthetic (GW501516, GW0742) ligands. In vivo, these PPARβ/δ agonists enhance the expression of FoxO1, a marker for muscle atrophy and metabolism, with a parallel increase in the rate-limiting pyruvate dehydrogenase kinase of glucose metabolism PDK4 [95]. Additionally, CD36 and lipoprotein lipase levels are also stimulated, suggesting a preferential use of fatty acids rather than glucose in muscle tissue in mouse and rat models [89]. Interestingly, PPARβ/δ and PPARα are also upregulated during fasting in the skeletal muscles of mice. However, in humans, PPARα protein levels are unaffected during fasting [96,97]. The expression level of the rate-limiting PDK4 enzyme, which inhibits the pyruvate dehydrogenase complex activity, is not affected in the PPARα KO mice under fasting conditions, suggesting that PPARβ/δ might be the primary factor that regulates fatty acid uptake and β-oxidation as an alternative source for energy production in skeletal muscle during fasting [70,89]. Mice treated with the PPARβ/δ agonist GW501516 also display increased expression levels of PGC-1α, together with an ameliorated wheel-running performance [98]. This treatment also increases succinate dehydrogenase (SDH)-positive myofibers, which are oxidative slow-twitch fibers rich in mitochondria, suggesting increased hydrolysis of triglycerides and catabolism of FAs through mitochondrial fatty acid β-oxidation [98]. 

In addition to the experimental use of agonists, tissue-specific PPARβ/δ gain and loss of function mouse models have been very instrumental for unveiling the skeletal muscle functions of this receptor isotype. Mice with muscle-specific overexpression of PPARβ/δ display increased fast/slow twitch oxidative myofibers, enhanced fatty acid catabolism in the muscles through mitochondrial β-oxidation, and decreased body fat mass with smaller adipocytes [99]. These transgenic mice display increased glucose metabolism in addition to increased fatty acid β-oxidation. Another transgenic mouse model expressing a constitutively active PPARβ/δ, the fusion protein VP16-PPARβ/δ, also displays decreased adiposity [100]. Moreover, muscle-specific overexpression of this constitutively active VP16-PPARβ/δ protein increases the number of SDH-positive myofibers in the hind limb muscles tibialis anterior and soleus of mice, suggesting a switch in substrate use in myofiber types from glycolysis to oxidation [100]. Additionally, in these transgenic mice, the myofiber type switch is associated with an increase in mitochondrial number and activity together with an increased blood capillary density, and myoglobin levels making the myofibers appear red in color. Most interestingly, these mice demonstrate increased performance by continuously running over twice the distance and time compared to control mice [100]. Thus, these transgenic mice with a muscle-specific constitutively active PPARβ/δ are more resistant to fatigue and present a phenotype that mimics the effects of endurance training [100]. Yet another transgenic model, expressing VP16-PPARβ/δ in adipose tissue, also displays increased fatty acid catabolism with decreased fat depots and better lipid profiles. These mice are also resistant to high fat diet induced obesity suggesting a plausible organ–organ crosstalk in these transgenic mice [101]. In a loss of function mouse model based on the ablation of PPARβ/δ in muscle only, impaired energy metabolism with decreased fast/slow twitch oxidative myofibers was observed [102]. Further, in these muscle-specific PPARβ/δ KO mice there also is an increase in body weight with both regular and high-fat-diet feeding. This increase in body weight is due to increased fat mass and adipocyte size in the white adipose tissues. Further, during aging these PPARβ/δ KO mice display adipocyte hypertrophy and glucose intolerance, and are insulin resistant, which is reminiscent of metabolic disorders such as obesity and diabetes in mice and humans [102]. Collectively, these findings have unveiled the crucial roles played by PPARβ/δ in skeletal muscle metabolism and indicate that loss of muscle oxidative function leads to diabetes and, in this case, not diabetes to muscle dysfunction. Additionally, *Pgc-1α* has been demonstrated to be a downstream target gene of PPARβ/δ in mice and humans [102,103]. Furthermore, PGC-1α expression increases in skeletal muscle during exercise and fasting [104,105] and overexpression of PPARβ/δ in rat muscles also increases PGC-1α protein levels with an impact on fatty acid oxidation and glucose metabolism [106]. Furthermore, mice overexpressing PGC-1α and PGC-1β in muscle phenocopy PPARβ/δ transgenic mice with increased oxidative myofibers [107,108].

We and others have reported previously that the deficiency of PPARβ/δ in mice results in a reduction in muscle satellite cell number and its regenerative capacity, suggesting that PPARβ/δ regulates postnatal myogenesis and regeneration in mice [109,110]. Thus, PPARβ/δ deficiency in mice ultimately results in muscle atrophy thereby resulting in decreased muscle and body weights [109]. In fact, several studies have implicated PPARβ/δ in postnatal myogenesis, myofiber switch to slow oxidative fibers, and oxidative metabolism in mitochondria [2,99,100,101,110]. Furthermore, activation in mice of PPARβ/δ with GW0742 generates muscle hyperplasia. The observed muscle hyperplasia was attributed to an increase in myonuclear density, which is associated with the enhanced expression of the muscle regulatory factors Myf5 and MyoD, suggesting their involvement in myocyte fusion. Interestingly, the decrease in myonuclear accretion during aging is blunted in mice treated with PPARβ/δ agonists [111,112]. In addition, MyoD, the master regulator of myogenesis, in association with the nuclear factor NF-kB transcription factor subunit RelB, occupies multiple sites on the *Pgc1β* promoter region in mice, which leads to an increased expression of *Pgc1β* and enhanced oxidative metabolism in the skeletal muscles [113].

PPARβ/δ KO mice mimic the effects of physical inactivity in humans. These mutant mice exhibit muscles with lower oxidative capacity, which leads to the development of obesity and diabetes, and reduced running capacity [102]. On the contrary, muscle-specific overexpression of PPARβ/δ and the use of PPARβ/δ synthetic agonists in mice phenocopies some of the effects of running in endurance training [98]. In fact, both endurance and resistance exercises result in increased PPARβ/δ expression in mouse muscles [99,114]. Furthermore, the administration of the AMPK activator 5-aminoimidazole-4-carboxamide-1-β-D-ribofuranoside (AICAR) and the GW0742 PPARβ/δ agonist potentiates the beneficial effects of exercise in mice [115]. The activation of PPARβ/δ also prevents inflammation and increases insulin sensitivity through the activation of AMPK and subsequent inhibition of the extracellular signal-regulated kinase ERK1/2 or lysophosphatidylcholines signaling [116,117]. Therefore, PPARβ/δ in myocytes is key for the maintenance of oxidative fibers and fiber-type switching. Altogether, PPARβ/δ contributes in a major way to muscle physiology and plasticity, and tissue homeostasis (Figure 2). It would be interesting to know if there are any compensatory effects of the other two PPAR isotypes in the muscle when there is a loss of PPARβ/δ. This question could be addressed by studying the muscle-specific knockout of PPARα, PPARβ/δ, and PPARγ and/or by analyzing double and triple PPAR KO mice. 

Constitutive overexpression of PPARγ in skeletal muscle of mice induces adiponectin production in muscle, decreases myosteatosis, and increases oxidative myofiber content and insulin sensitivity [118]. On the contrary, muscle-specific PPARγ KO mice display an increase in adipose tissue mass, glucose intolerance, and insulin resistance, but these mice still respond to thiazolidinediones [119,120]. The PPARγ coactivators (PGC-1α and PGC-1β) are also expressed in skeletal muscle and are involved in mitochondrial biogenesis [105]. The administration of synthetic PPARγ agonists in mice lacking PPARγ in adipose tissue increases insulin sensitivity in liver and skeletal muscle despite increasing circulating triglyceride levels suggesting that these drugs have hypoglycemic effects not dependent on adipose tissue PPARγ [121]. However, activation of PPARγ in muscle increases glucose use through GLUT1 and GLUT4 activation [122] (Figure 2). Together, the above suggests that all three PPAR isotypes are involved in skeletal muscle lipid and glucose metabolism. Moreover, the activation of PPARα, PPARβ/δ, and PGC-1α and -1β phenocopy some of the benefits of exercise. 

## 4. PPARs in Muscle Wasting

The attention given to PPARs has also gained importance due to their role in muscle pathophysiology associated with the metabolic syndrome, myopathies, muscular dystrophies, cancer cachexia, aging, and respiratory and cardiovascular diseases [88,89]. The synthetic PPARα agonists such as gemfibrozil, bezafibrate, and fenofibrate are used to treat cardiovascular diseases as potent hypolipidemic drugs [89]. Fibrates lower the circulating triglyceride levels in the blood by inducing hepatic fatty acid oxidation and reducing the apolipoprotein apoC3 expression levels, and increasing the expression levels of high-density lipoproteins through an increase in apo-A1 and -A2 levels [88]. The fibrates may also have a hypoglycemic anti-diabetic effect as a consequence of their hypolipidemic action that warrants further studies. Further, the use of the pan-PPAR ligand bezafibrate improves fatty acid oxidation defects in cultures of human fibroblasts deficient in CPT-2 and the very long chain acyl-CoA dehydrogenase (VLCAD) [123,124]. Interestingly, the activation of PPARβ/δ ameliorates the human Duchenne muscular dystrophy (DMD) phenotype in X-linked muscular dystrophy (mdx) mice that have a spontaneous mutation in the dystrophin gene. PPARβ/δ regulates Utrophin A by directly binding to the PPRE in the Utrophin A promoter region [125]. In these mdx mice, the upregulation of Utrophin A compensates dystrophin deficiency and, thereby, myofiber loss. Further, the activation of PPARβ/δ by synthetic agonists enhances calcineurin-dependent remodeling of myofibers in the mdx mice [125]. The calcineurin- and nuclear factor of activated T-cells (NFAT)-dependent pathway regulates *Myf5* gene expression in an in vitro model of myotubes reserve population of stem cells [126]. Further, the expression of Myf5 is transiently increased by the pharmacological activation of PPARβ/δ [126]. The calcineurin/NFAT signaling-dependent activation of Utrophin A also ameliorates the DMD phenotype in mdx mice [127]. The co-administration of cyclosporine A with a PPARβ/δ agonist in mice inhibits the myofiber switch, suggesting an indirect effect of the calcineurin pathway [111]. Interestingly, it has also been shown that cardiac angiogenesis and myocyte growth are mediated, at least in part, by transcriptional activation of calcineurin by ligand-activated PPARβ/δ [128]. Together, these observations indicate that activation of PPARβ/δ by synthetic ligands might have potential for the treatment of muscular dystrophies such as DMD.

Furthermore, synthetic PPARγ agonists such as rosiglitazone and pioglitazone are used in type 2 diabetic patients for their potent antidiabetic effects [89]. More recently, it has been shown that rosiglitazone treatment of in vitro cocultured insulin-resistant mouse 3T3-L1 adipocytes and C2C12 myotubes improved the insulin resistance in C2C12 myotubes, suggesting an organ–organ crosstalk between the adipocytes and skeletal muscle in type 2 diabetes [129]. However, the direct link between PPARγ and glucose homeostasis in skeletal muscle has not been known likely because of the low level of expression of this receptor. The RXR agonist LG100268 and the heterodimeric partner PPARγ agonist troglitazone have also been tested against diabetes for their potent hypoglycemic effect in the human myotubes culture model [130].

Mesenchymal stromal cells (MSC) may provide a source of cells to treat muscle diseases such as DMD. In light of the role of Wnts (wingless-type) in myogenesis during embryogenesis and muscle repair, it was hypothesized that the Wnt pathway could be implicated in the myogenic differentiation of MSC [131]. It was indeed demonstrated that overexpression of activated β-catenin in MSC stimulated myogenic differentiation of these cells, and suppressed their adipogenic differentiation via downregulating the expressions of PPARγ and the CCAAT/enhancer binding protein C/EBPα [131]. More recently, it has also been demonstrated that PPAR agonists stimulate bone marrow mesenchymal stem cells to enter either the myogenic and adipogenic lineages [132]. Along the same line of experimentation, culture conditions have been established to obtain pure mesenchymal precursors from human embryonic stem cells, which can be differentiated into skeletal muscle cells, adipocytes, cartilage, and bone [133]. Interestingly, PPARγ and its ligands also promote osteoclast differentiation and bone resorption [134,135,136]. Taken together, these findings on the roles of PPARs in MSC differentiation could inspire new pharmacological approaches to generate different mesenchymal cell types for potential future clinical applications. Although there is a long way to go before implementing cell therapy, these works provide a basis for stem-cell-based tissue repair interventions.

## 5. PPAR Interactions with the Gut Microbiota

**Figure 3:**
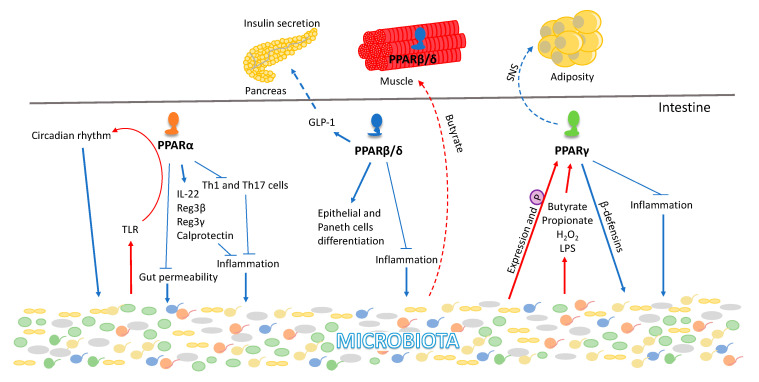
Roles of PPARs in the gastrointestinal tract. All three PPAR isotypes act in the intestine to reduce inflammation, whereby they modulate the microbiota composition. PPARα functionally interacts with gut bacteria and mediates their signals to regulate the circadian rhythm. Intestinal PPARβ/δ plays an important role in the development of the intestine, whereas in muscle it is activated by bacterial metabolites, such as short-chain fatty acids like butyrate. Besides its key role of extinguishing inflammation and its dialog with gut bacteria, PPARγ also mediates information concerning nutrient status from the gut to the adipose tissue. Circled P indicates phosphorylation. Red and blue arrows indicate microbiota and host effects, respectively. Solid lines represent the reciprocal interactions of PPARs in the intestines and microbiota. Dotted lines show the distant effects of intestinal PPARs and organ-organ crosstalk on various metabolic organs such as pancreas, muscle and white adipose tissue. TLR: toll-like receptors; LPS: lipopolysaccharides; SNS: sympathetic nervous system; Reg3: regenerating islet-derived 3; GLP-1: glucagon-like peptide-1; Th1, 17 cells: T-helper type 1, 17 cells; IL-22: interleukin-22.

The gastrointestinal tract harbors a wealth of microorganisms, viz., bacteria, archaea, protozoa, yeast, and fungi. The gut microbial population is defined by high composition variability in different hosts. In humans, the individual bacterial makeup is determined by various factors from childbirth to geriatrics, gender, lifestyle, immunization, antibiotic use, demography, and diet [137,138,139,140]. Microbiota dysbiosis is associated with multiple disorders, such as inflammatory bowel disease (IBD), irritable bowel syndrome (IBS), non-alcoholic fatty liver disease (NAFLD), and type 2 diabetes (T2D). All three PPARs show distinct ways of interacting with the microbiota, mostly in the context of inflammation and metabolism [54] (Figure 3). PPARs are expressed along the gastrointestinal tract with an individual expression pattern from the duodenum to the distal colon [141,142]. The highest PPARα expression is in the proximal intestine and its levels decrease with a lowest expression in the colon [68]. The PPARα KO mice phenotype is associated with dysbiosis caused by an increase in the number of intestinal T_h_1 and T_h_17 (T-helper) cells and regulation of the expression levels of interleukin IL-22 as well as the antimicrobial peptides of the regenerating family members Reg3β, Reg3γ, and calprotectin [143]. Consistently, PPARα is associated with the anti-inflammatory response of the intestine to commensal microbiota activity. It protects the intestine from colitis-induced permeability by preventing neutrophil infiltration [144,145,146,147]. Furthermore, PPARα is a major contributor to functional circadian rhythm in the gut, thereby affecting the body’s chrono-metabolism [148,149,150], whereas gut dysbiosis alters the circadian rhythm. Importantly, in intestinal epithelial cells, microbiota signals to PPARα via toll-like receptors (TLR) and consequently contributes to the regulation of expression of intestinal circadian genes [151]. Moreover, alteration of the circadian rhythm also affects the gut-microbiota composition, revealing a two-way dialog between the microbiota and its host. 

PPARβ/δ is highly expressed in the gastrointestinal tract except for the colon and is involved in the proliferation and differentiation of intestinal epithelial cells, paneth cells differentiation, and tissue homeostasis [142,152,153]. Intestinal PPARβ/δ shows anti-inflammatory properties in IBD and experimental colitis [153,154,155]. PPARβ/δ induces the glucagon-like peptide GLP-1 production in enteroendocrine L-cells after food consumption, thereby modulating pancreatic β-cell function [156] and translating microbial signals to peripheral organs. Recent studies have highlighted an impact of the interactions of gut microbiota and PPARs in skeletal muscle physiology and pathology [157,158,159,160,161] (Figure 3). Butyrate, a product of bacterial fiber fermentation, stimulated the expression level of PPARβ/δ in the skeletal muscle of mice and in rat L6 myoblasts in vitro [162]. We have also demonstrated that treatment of mice with the antibiotic drug metronidazole leads to an increase in proteobacteria and results in skeletal muscle atrophy [142].

The gastrointestinal tract shows the second-highest expression levels of PPARγ after adipose tissue [68,163]. So far, the role of PPARγ expression in the intestine has largely been explored for its anti-tumorigenic role, which is evidenced by its regulation of cell proliferation, differentiation, and apoptosis [164,165,166]. However, PPARγ also acts as an anti-inflammatory factor and is responsible for the selective killing of bacteria associated with IBD by maintaining constitutive epithelial expression of a subset of beta-defensin in the colon [167] (Figure 3). Consequently, its agonists have been applied to mitigate the symptoms of IBD, ulcerative colitis, and Crohn’s disease [168,169,170,171]. Colonic mucosa of PPARγ KO mice is defective in killing several major strains of the intestinal microbiota, including *Candida albicans*, *Bacteroides fragilis*, *Enterococcus faecalis*, and *Escherichia coli* [165]. Moreover, PPARγ maintains an anaerobic condition in the large intestine limiting the inducible nitric oxide synthase (iNOS) production from butyrate through β-oxidation, facilitating the growth of facultative anaerobes [172]. However, various commensal bacteria belonging to *Firmicutes*, *Bacteriodetes*, *Fusobacteria*, and *Actinobacteria* also modulate PPARγ expression and activity [173]. Diverse bacterial byproducts and metabolites such as butyrate, propionate, H_2_O_2_, and lipopolysaccharides impact the expression and/or activity of PPARγ [173,174,175,176]. Bacterial strains including *Enterococcus faecalis*, *Roseburia hominis, Roseburia intestinalis, Fusobacterium naviforme*, and *Streptococcus salivarius* influence the phosphorylation status of PPARγ and thereby its transcriptional activity [173,177,178]. In the mouse model of experimental colitis, *Lactobacillus paracasei* B21060 induces the expression of PPARγ and β-defensin, which promotes intestinal homeostasis. Butyrate and propionate produced by *Akkermansia municiniphila* modulate the expression levels of PPARγ and its downstream target gene angiopoietin-like protein-4 (*Angptl4*) [142]. However, *Akkermansia municiniphila* is associated with decreased expression of PPARγ in mouse organoids [179]. Furthermore, we have shown that intestinal PPARγ regulates body fat mass by signaling through the sympathetic nervous system [180]. Thus, intestinal PPARγ activation by nutrients and bacterial metabolites can impact the adipose tissues. Together, this suggests that the expression level of PPARγ in the intestines can be modulated by gut microbial species and, in turn, PPARγ can alter the gut microbiota composition leading to an impact at the whole-body level. Thus, it is conceivable, but remains to be demonstrated, that all PPARs are influenced by the gut-microbiota composition in maintaining the intestinal homeostasis (Figure 3). 

## 6. PPARs and the Gut-Muscle Axis

Recently, the importance of the gut–muscle axis has been underscored by several research groups [181,182,183,184,185,186,187,188]. However, the causal connection between gut dysbiosis and musculoskeletal disorders and diseases are not known yet. Compared to specific-pathogen-free (SPF) mice, the whole-body mass was found to be higher in germ-free (GF) mice and in mice treated with the antibiotics beta-lactum and macrolide, suggesting a link between the gut microbiome and muscle [189,190]. This increase in the whole-body weight can be attributed, at least in part, to the increased cecum weight in these GF mice. GF mice display skeletal muscle atrophy with a decrease in muscle growth factors such as insulin-like growth factor 1 (IGF1), the amino acids alanine and glycine, and in mitochondrial function [157]. However, the GF mice colonized with fecal samples of SPF mice displayed an increase in their muscle mass and oxidative capacity and decrease in muscle atrophy [157]. Additionally, the mouse grip strength and endurance exercise capacity were reduced in GF mice as compared to SPF mice [157]. Germ-free mice colonized with fecal samples of highly physically active adults increased their grip strength as compared to adults with low physical activity; however, their whole-body lean mass and endurance capacity were not changed [157]. These observations suggest that additional factors associated with or produced by exercise and the enrichment of *Barnesiella intestinihominis* might promote a higher physical activity of older adults with lean body mass when compared to adults with low physical performance [158]. The antibiotic-treated mice showed increased fatigue, decreased treadmill running endurance exercise capacity, and reduced muscle mass [159,160,161]. Interestingly, the muscle endurance capacity can be regularized by natural bacterial reseeding [160]. Aging causes an alteration in the gut microbiota composition such as an increase in proteolytic bacteria and a decrease in saccharolytic bacteria, which are associated with sarcopenia [139,191]. Oral gavaging of *Lactobacillus casei* or *Bifidobacterium longum* in aged mice increases muscle mass and grip strength [192]. Further, colonization by *Eubacterium rectal*, *Lactobacillus plantarum,* and *Clostridium coccoides* increases the energy metabolism in GF mice [193]. Inoculation of *Veillonella atypica* obtained from the fecal samples of marathon runners into mice enhanced their treadmill running capacity [194]. GF mice transplanted with obese pig microbiota develop increased lipogenesis and fat mass with decreased fast glycolytic type 2b myofibers in gastrocnemius muscle, when compared to microbiota transplants coming from lean pig microbiota [195]. Further, GF mice fed with the short-chain fatty acids acetate, propionate, and butyrate increased muscle mass and function [157]. In vitro, the differentiated human skeletal myoblasts LHCN-M2 treated with the gut microbial metabolite isovanillic acid 3-O-sulfate that circulates in the blood increased glucose metabolism through GLUT1/4-PI3K-Akt signaling [196]. In aged people with higher muscle strength, there is an increase in *Prevotellaceae*, *Prevotella*, and *Barnesiella* compared to people with lower muscle strength. Additionally, a 12-week of endurance exercise in aged people promotes an increase in *Bacteroids* species [158]. However, further detailed investigations are needed to better elucidate the gut–muscle axis and its functions in aged individuals, and in muscle pathophysiology.

Recently, we have shown that metronidazole-treated mice present gut dysbiosis and skeletal muscle atrophy [159]. Further, in these mice treated with metronidazole, there are changes associated with the muscle peripheral circadian clock machinery and metabolic regulators such as PPARγ, suggesting a possible link between gut dysbiosis and the observed muscle chrono-metabolism phenotype [159]. Interestingly, both PPARγ and its target gene adiponectin were significantly upregulated in the skeletal muscle of metronidazole-treated SPF mice, which might enhance muscle fatty acid uptake and insulin sensitivity effects, however, that remains to be demonstrated. Furthermore, the peripheral core clock repressor proteins cryptochrome Cry1 and Cry2 modulate PPARβ/δ in skeletal muscle through AMPK-dependent signaling, suggesting that PPARβ/δ is a downstream target gene of the circadian clock machinery possibly as part of its involvement in chrono-metabolism [159]. 

## 7. Conclusions

Even though all three PPAR isotypes are expressed in skeletal muscle to varying degrees, the roles of the individual PPARs or their complementary and/or compensatory effects are still not well-known. The predominantly expressed PPARβ/δ isotype in skeletal muscle has been widely studied both in vivo and in vitro. PPARβ/δ acts as a key regulator of glucose metabolism and in promoting the uptake of lipids and their use as an energy source during fasting and exercise. Pharmacological activation of PPARβ/δ in skeletal muscles also phenocopies some of the benefits of physical exercise in muscle remodeling with a myofiber switch to oxidative phenotype and alleviation of muscle disorders. However, very little is known about the other two PPAR isotypes, i.e., PPARα and PPARγ, in both healthy muscle physiology and in pathological conditions. Furthermore, the effects of agonists in muscle remain to be clarified as they also have effects in other organs and the whole organism. Recently, the gut–muscle axis has emerged and causal connections between PPARs and muscle pathophysiology and chrono-metabolism are being progressively unveiled. Present knowledge suggests that much more work is needed in skeletal muscle health and disease for designing lifestyle interventions and therapies, even though it is well-established that PPARs play an important role in regulating energy metabolism, inflammation, and circadian rhythm. The use of single- and multiple-tissue-specific KO models for PPARs will contribute to the gaining of valuable additional knowledge on the central contribution of these receptors in muscle.

## Abbreviations

AICAR	5-Aminiimidazole-4-carboxamide ribonucleotide
Akt	Protein kinase B
Ampk	AMP-activated protein kinase
Angptl4	Angiopoietin-like 4
ApoC3	Apolipoprotein C3
ATP	Adenosine Triphosphate
CD36	Cluster of differentiation 36
C/EBP	CCAAT/enhancer binding protein
Cox10	Cytochrome oxidase 10
CPT1/2	Carnitine palmitoyltransferase½
Cry1/2	Cryptochrome ½
DMD	Duchenne muscular dystrophy
FA	Fatty acid
FABP3	Fatty acid binding protein 3
FoxO1	Forkhead box O1
GLP-1	Glucagon-like peptide-1
GLUT1/4	Glucose transporter ¼
HGF	Hepatocyte growth factor
H_2_O_2_	Hydrogen peroxide
IGF1	Insulin-like growth factor
IBD	Inflammatory bowel disease
IBS	Irritable bowel syndrome
IL-22	Interleukin-22
iNOS	Inducible nitric oxide synthase
KO	Knockout
LPL	Lipoprotein lipase
LPS	Lipopolysaccharide
mdx	Muscular dystrophy X-linked
Mef2	Myocyte enhancer factor 2
MRF4	Myogenic regulatory factor 4
MyoD	Myogenic differentiation
Myf5	Myogenic factor 5
NAFLD	Nonalcoholic fatty liver disease
NFAT	Nuclear factor of activated T-cells
NR1C1-3	Nuclear receptor 1C-1-3
Pax3/7	Paired box 3/7
PDK4	Pyruvate dehydrogenase kinase 4
PGC1	PPAR gamma coactivator 1
PI3K	Phosphoinositide 3-kinase
PPAR	Peroxisome proliferator-activated receptor
PPRE	Peroxisome proliferator response element
Reg3	Regenerating islet-derived protein 3
SDH	Succinate dehydrogenase
SF	Scatter factor
SNS	Sympathetic nervous system
T2D	Type 2 diabetes
Th1/17	T-helper cells 1/17
TLR	Toll-like receptor
VLCAD	Very long-chain acyl-CoA dehydrogenase
Wnt	Wingless-type MMTV integration site family
